# AFK-PD alleviated osteoarthritis progression by chondroprotective and anti-inflammatory activity 

**DOI:** 10.3389/fphar.2024.1439678

**Published:** 2024-08-29

**Authors:** Zhuang Qian, Jie Xu, Lei Zhang, Qian Deng, Zhenlin Fan, Xueqiang Guo, Zhuo Liang, Weiyun Wang, Lei Wang, Xiaohua Liao, Wenjie Ren

**Affiliations:** ^1^ Clinical Medical Center of Tissue Engineering and Regeneration, Institutes of Health Central Plain, The Third Affiliated Hospital of Xinxiang Medical University, Xinxiang Medical University, Xinxiang, China; ^2^ Department of Rheumatology and Immunology, Nanjing Drum Tower Hospital, The Affiliated Hospital of Nanjing University Medical School, Nanjing, China

**Keywords:** AFK-PD, osteoarthritis, chondrocyte, inflammation, MAPK/ NF-κB pathways

## Abstract

Osteoarthritis (OA) is the most prevalent cartilage degenerative and low-grade inflammatory disease of the whole joint. However, there are currently no FDA-approved drugs or global regulatory agency-approved treatments OA disease modification. Therefore, it’s essential to explore novel effective therapeutic strategies for OA. In our study, we investigated the effects of AFK-PD, a novel pyridone agent, on the development of OA induced by destabilization of the medial meniscus (DMM) *in vivo*, and its impact on the function of chondrocytes treated with IL-1β *in vitro*. Our results demonstrated AFK-PD alleviated OA progression through inhibiting cartilage degeneration, articular inflammation and osteophyte formation. Notably, AFK-PD inhibited chondrocyte inflammation and synovial macrophage M1 polarization, leading to the attenuation of articular inflammation. Additionally, AFK-PD promoted chondrocyte anabolism while mitigating catabolism and apoptosis, effectively inhibiting cartilage degeneration. Mechanistically, AFK-PD suppressed the expression of key signaling molecules involved in the MAPK pathway, such as p-ERK1/2 and p-JNK, as well as the NF-κB signaling molecule p-p65, in IL-1β-induced chondrocytes. These findings suggest AFK-PD ameliorates the development of OA by protecting chondrocyte functions and inhibiting articular inflammation in chondrocytes and synovial macrophages. Overall, our study highlights AFK-PD as a promising therapeutic candidate for the treatment of OA.

## 1 Introduction

OA is the most prevalent chronic musculoskeletal disease characterized by pain and disability. It is widely recognized that OA is a whole joint disease characterized by cartilage degeneration, synovial inflammatory (synovitis), osteophyte formation and subchondral bone remodeling. Synovial and articular inflammatory environment within the joint are the key factors for chondrocyte apoptosis and cartilage degeneration. Specifically, degraded cartilage matrix releases damage-associated molecular patterns (DAMPs), which trigger inflammatory responses of chondrocytes and synovial macrophages by secreting proinflammatory cytokines (including IL-1β, TNF-α and IL6) and matrix-degradative enzymes such as matrix metalloproteinases (MMPs) (Zhang et al., 2020; Hashizume et al., 2024). Nowadays, conventional non-steroidal anti-inflammatory drugs (NSAIDs) are widely used to alleviate joint inflammation. However, they only provide symptomatic relief without improving cartilage degeneration and are associated with inevitable side effects (Zhou et al., 2019). Therefore, it’s important to explore novel effective therapeutic strategies for OA.

IL-1β is a major inducer of chondrocytes inflammation and metabolism imbalance. When exposed to IL-1β inflammatory stimuli, catabolic factors (MMP13) are increased whereas anabolic factors (Col2a1, Acan and Sox9) are decreased. This imbalance in chondrocyte metabolism and the subsequent apoptosis ultimately lead to cartilage degradation (Wang et al., 2023). A large number of research have confirmed MAPK and NF-κB signaling pathways were activated in OA cartilage and IL-1β-induced mouse chondrocytes (Saklatvala, 2007). In detail, during OA progression, inflammatory mediators (such as IL-1β) induce phosphorylation of p38, JNK, and ERK1/2, the key factors of in MAPK signaling pathway, and translocation of these phosphorylated factors to the nucleus in chondrocytes. In addition, inflammatory mediators also activate the phosphorylation and nucleus translocation of NF-κB p65, the key factor in NF-κB signaling pathway, in chondrocytes. These phosphorylated factors further lead to the release of pro-inflammatory cytokines, metaloproteinases (MMPs) and aggrecanases, which shift chondrocytes metabolism towards a catabolic state, ultimately leading to chondrocytes apoptosis and cartilage matrix degeneration (Yan et al., 2020; Gratal et al., 2022; Lu et al., 2023). So, targeting inflammation-associated factors and signaling pathways holds promise as alternative and innovative therapies.

AKF-PD (1-(3-fluorophenyl)-5-methyl-2-(1H)-pyridone), referred to as Fluorofenidone, is a novel low-molecular-weight pyridone agent. Increasing evidence has demonstrated AFK-PD possesses various pharmacological properties, including anti-inflammation, anti-apoptosis and anti-oxidative in conditions such as liver fibrosis, liver failure, kidney injury and lung injury (Jiang et al., 2019; Lv et al., 2021; Tu et al., 2021; Gu et al., 2023). Recently, it has been shown that Pirfenidone, an analogue of AFK-PD, attenuated OA progression by inhibiting synovial fibrosis and inflammation (Wei et al., 2021). In addition, Many of studies have uncovered AFK-PD had anti-inflammatory and anti-apoptotic effects by restraining MAPK and NF-κB pathways in many of diseases such as liver fibrosis, kidney injury and lung injury (Peng et al., 2013; Qin et al., 2015; Tang et al., 2015; Jiang et al., 2019; Peng et al., 2019; Lv et al., 2021; Tu et al., 2021; Gu et al., 2023). However, it’s unclear whether AFK-PD ameliorates OA progression by regulating chondrocyte inflammation and metabolism, as well as the MAPK and NF-κB signaling pathways involved in such fine-tuned regulation.

In this study, we aimed to investigate the impact of AFK-PD on the progression of OA and elucidate the underlying mechanism by which AFK-PD regulates inflammation and chondrocyte metabolism in IL-1β-induced mouse chondrocytes. Our findings demonstrated that AFK-PD effectively inhibited synovial and chondrocytes inflammation and shifted chondrocytes catabolic to anabolic metabolism via mitigating MAPK/NF-κB signaling. Ultimately, these effects resulted in the amelioration of OA progression.

## 2 Materials and methods

### 2.1 Primary chondrocytes extract and treatment

Primary chondrocytes were isolated from the femoral condyles and tibial plateau of 3-day-old mice, following the previously described methods (Salvat et al., 2005; Gosset et al., 2008). Briefly, the mice were euthanized and sterilized with 75% ethanol for 2 min. The articular cartilage was then isolated from the femoral condyles and tibial plateau under a dissecting microscope. Subsequently, the articular cartilage was incubated in 0.2% collagenase (C5138, Sigma-Aldrich, Missouri, United States) for 30 min at 37°C. After three washes with PBS, the articular cartilage was incubated in 0.2% collagenase for an additional 3 h at 37°C. The resulting cell suspension was aspirated repeatedly and filtered through a 100-μm cell strainer. The cells were then rinsed in PBS, counted, and seeded in 6-well plates at a density of 1 million cells per well in DMEM (11965092, Thermo Fisher Scientific, Massachusetts, United States) supplemented with 100 units/mL penicillin, 100 μg/mL streptomycin, 50 μg/mL ascorbic acid, and 10% fetal bovine serum (A5670701, Thermo Fisher Scientific, Massachusetts, United States). The chondrocytes were subsequently treated with recombinant IL-1β (10 ng/mL; P06804, R&D Systems, Minnesota, United States) and AFK-PD (Provided by Professor Lijian Tao from Central South University) for 24 h.

### 2.2 Cell viability

Cell viability was assessed by Cell Counting Kit-8 (BS350B, Biosharp, Wuhan, China) following the manufacturer’s instructions. Primary chondrocyte (8×10^3^/well) seeded in 96-well plates were exposed to AFK-PD at various concentrations for 48 h. Subsequently, the absorbance was recorded at 450 nm using a microplate reader (Bio-Rad, Hercules, CA, United States) (Lou et al., 2023).

### 2.3 Knee osteoarthritis model

The adult C57/BL6 mice were purchased from Beijing Vital River Laboratory Animal Technology Co. Ltd.

Osteoarthritis was established in 8-week-old male mice by destabilizing the medial meniscus (DMM) following previous studies (Glasson et al., 2007). Briefly, mice firstly were anesthetized with isoflurane (1349003, Sigma-Aldrich, Missouri, United States). The right knee was then subjected to the transection of the medial meniscotibial ligament under a dissecting microscope. The sham operation was only subjected with medial capsulotomy in right knee. The mice that underwent the DMM procedure were randomly divided into two groups (n = 8). One week after the operation, AFK-PD treated group received an intra-articular injection of 8 μL AFK-PD (dissolved in saline at a concentration of 400 μg/mL). The control group was injected with saline. In the sham group, mice were injected with the same volume of saline (n = 8). All group were administered intra-articular injection once a week for 7 weeks.

All animal studies were authorized and conducted in accordance with the Animal Care and Use Committee of Xinxiang Medical University.

### 2.4 Histological analysis

After 8-week OA surgery, mice were sacrificed and the right knee joints were fixed in 4% paraformaldehyde. Subsequently, decalcification was performed using 10% EDTA for 4 weeks, and the joints were embedded in paraffin. Coronal sections with a thickness of 4 μm were obtained through the knee joints. These sections were stained with Safranin O/Fast Green (G1371, Solarbio, Nanjing, China) according to the recommended protocol. Histologic changes of articular cartilage were scored using recommended Osteoarthritis Research Society International (OARSI) (cartilage OA histopathology scoring system, on a scale of 0–6) (Glasson et al., 2010). Additionally, the sections were stained with hematoxylin and eosin (H&E) (G1120, Solarbio, Nanjing, China) to assay joint synovitis using synovitis scoring system (Gerwin et al., 2010).

All slides were evaluated independently by two investigators who were blinded to the treatment regimen.

### 2.5 Micro-computed tomography (micro-CT)

Mice knee joints were fixed in 4% PFA, and subsequently, the microstructure of the joints was analyzed using a micro-CT scanner (mCT80; Scanco Medical AG) as described (Li et al., 2022). The three-dimensional (3D) reconstruction images of the joints were obtained using Scanco Medical software.

### 2.6 Immunohistochemistry

Immunohistochemical staining was performed using the DAB staining method according to the recommended protocol. Briefly, after deparaffinization and rehydration, antigen retrieval was carried out using 2.5 mg/mL trypsin for 40 min. The sections were then treated with 3% H_2_O_2_ for 10 min to block endogenous peroxidase activity. Subsequently, after blocking with 5% BSA (37,520, Thermo Fisher Scientific, Massachusetts, United States) for 1 h at 37°C, the sections were incubated overnight at 4°C with the primary antibody. On the following day, the sections were incubated with HRP-labeled secondary antibodies for 1 h at 37°C. The protein expression signal was visualized as a brown reaction product using the peroxide substrate 3,3′-diaminobenzidine (DAB) (ZLI-9017, ZSGB-BIO, Beijing, China), and counterstained with hematoxylin. The number of stained cells was counted in five random high-magnification fields within the articular cartilage by three investigators who were blinded to the treatment regimen. The average percentage of positive cells to total cells was calculated.

### 2.7 Immunofluorescence staining

Immunofluorescence staining was performed on 4 μm paraffin sections. Briefly, after deparaffinization, rehydration, and antigen retrieval, the sections were incubated overnight at 4°C with the indicated primary antibodies. Subsequently, the sections were incubated with fluorochrome-labeled secondary antibodies (Fluor 488 or TRITC) (115-025-003 and 115-545-003, Pennsylvania, United States) at 37°C for 1.5 h. Nuclei were stained with 4,6-diamidino-2-phenylindole (DAPI) (P0131, Beyotime Biotechnology, Shanghai, China) for 15 min at room temperature. Images were captured using a fluorescence microscope (Nikon Eclipse Ti-S, Tokyo, Japan). The number of positive cells was quantified in five random high-magnification fields within the articular cartilage by three investigators who were blinded to the treatment regimen. The average percentage of positive cells to total cells was calculated.

### 2.8 Chondrocytes micro-mass culture and alcian blue staining

The 20 μL suspension containing primary 2 × 10^5^ chondrocytes in DMEM medium was dropped into each well of 24-well plate. After 2 h, micro-masses were treated with IL-1β and AFK-PD in DMEM with 10% FBS for 7 days. Alcian blue staining was performed with Alcian Blue Stain Kit (G1565, Solarbio, and Beijing, China) according to the recommended protocol. The micro-masses were washed with PBS, fixed with paraformaldehyde for 10 min, rinsed with 0.1 N HCl, and then stained with 1% alcian blue at room temperature for 30 min (Atsuta et al., 2019).

### 2.9 RAW264.7 cells culture

RAW264.7 cells were obtained from the Cell Bank of Type Culture Collection of Chinese Academy of Science (Shanghai, China) and cultured in DMEM supplemented with 10% fetal bovine serum and 100 units/mL penicillin and 100 μg/mL streptomycin at 37°C and 5% CO_2_ condition. 1 × 10^5^ RAW264.7 cells were polarized to M1 macrophage with 50 ng/mL lipopolysaccharide (LPS) (#L2630, Sigma-Aldrich, St. Louis, MO, United States), and subsequently treated with AFK-PD (400 μg/mL for 24 h to detect the mRNA level of M1-related markers).

### 2.10 TUNEL assay

Apoptotic cells from articular cartilage and primary chondrocytes were detected by *In Situ* Cell Death Detection Kit (No.12156792910, Roche, Mannheim, Germany), according to the manufacturer’s instructions. TUNEL-labeled cells visualized as red fluorescence, while nuclei were counterstained with DAPI. The percentage of TUNEL-positive cells was calculated as the number of labeled cells/total cells per high-magnification field. All determinations were made by the same observer blinded to the treatment category.

### 2.11 Western blot

The cells were washed with chilled PBS and lysed in a lysis buffer. The lysates were then subjected to 10% sodium dodecyl sulfate-polyacrylamide gel electrophoresis (SDS-PAGE) and transferred to polyvinylidene difluoride (PVDF) membranes. Following transfer, the membranes were incubated overnight at 4°C with the respective primary antibodies as indicated. The next day, the membranes were incubated with secondary antibodies for 60 min. Subsequently, the protein bands were visualized using an enhanced chemiluminescence detection system (WBKLS0100, Millipore, Burlington, United States). The resulting bands were quantified using the ImageJ software through densitometry analysis (Qian et al., 2023).

### 2.12 RNA extraction and quantitative real-time PCR

Total RNA was extracted from cells using RNA-Quick Purification Kit (RN001, ES Science, Shanghai, China). The isolated RNA was reverse transcribed using HiScript Ill 1st Strand cDNA Synthesis Kit (R312-02, Vazyme, Nanjing, China) to synthesize cDNA. Real-time quantitative PCR was carried out in a MJ Mini Real-Time PCR Detection System using Taq Pro Universal SYBR qPCR Master Mix (Q712-02, Vazyme, Nanjing, China). Gene expression was normalized to GAPDH, and relative expression was calculated using the 2^–(ΔΔCt)^ method. The following primer sequences were described in Supplementary Table S2.

### 2.13 Statistical analysis

All the data were presented as the mean ± SD. Data analysis was conducted using PASW Statistics 17 (SPSS Inc.). And statistical significance was determined by an unpaired, two-tailed Student t test between the 2 groups or one-way ANOVA for more than 2 groups. Values of *p* < 0.05 were considered statistically significant.

## 3 Results

### 3.1 Effect of AFK-PD on metabolism and apoptosis of IL-1β-induced chondrocytes

Firstly, the cytotoxicity of AFK-PD on mouse primary chondrocytes was tested using CCK-8. The results showed that AFK-PD had no cytotoxicity at concentration of 0–400 μg/mL (Supplementary Figure S1). Furthermore, we explored the protein expression of MMP13, a catabolic marker for chondrocytes, in primary IL-1β-induced chondrocytes treated with AFK-PD at concentration of 0–400 μg/ml. As shown in Supplementary Figure S2, AFK-PD at concentrations of 200 and 400 μg/mL significantly inhibited the MMP13 expression in IL-1β-induced chondrocytes. Based on many of evidence confirming the effective concentration of AFK-PD to be 400 μg/mL in different kinds of cells (Jiang et al., 2019; Lv et al., 2021; Tu et al., 2021; Gu et al., 2023), subsequent experiments involving AFK-PD treatment were performed at this concentration.

**FIGURE 1:**
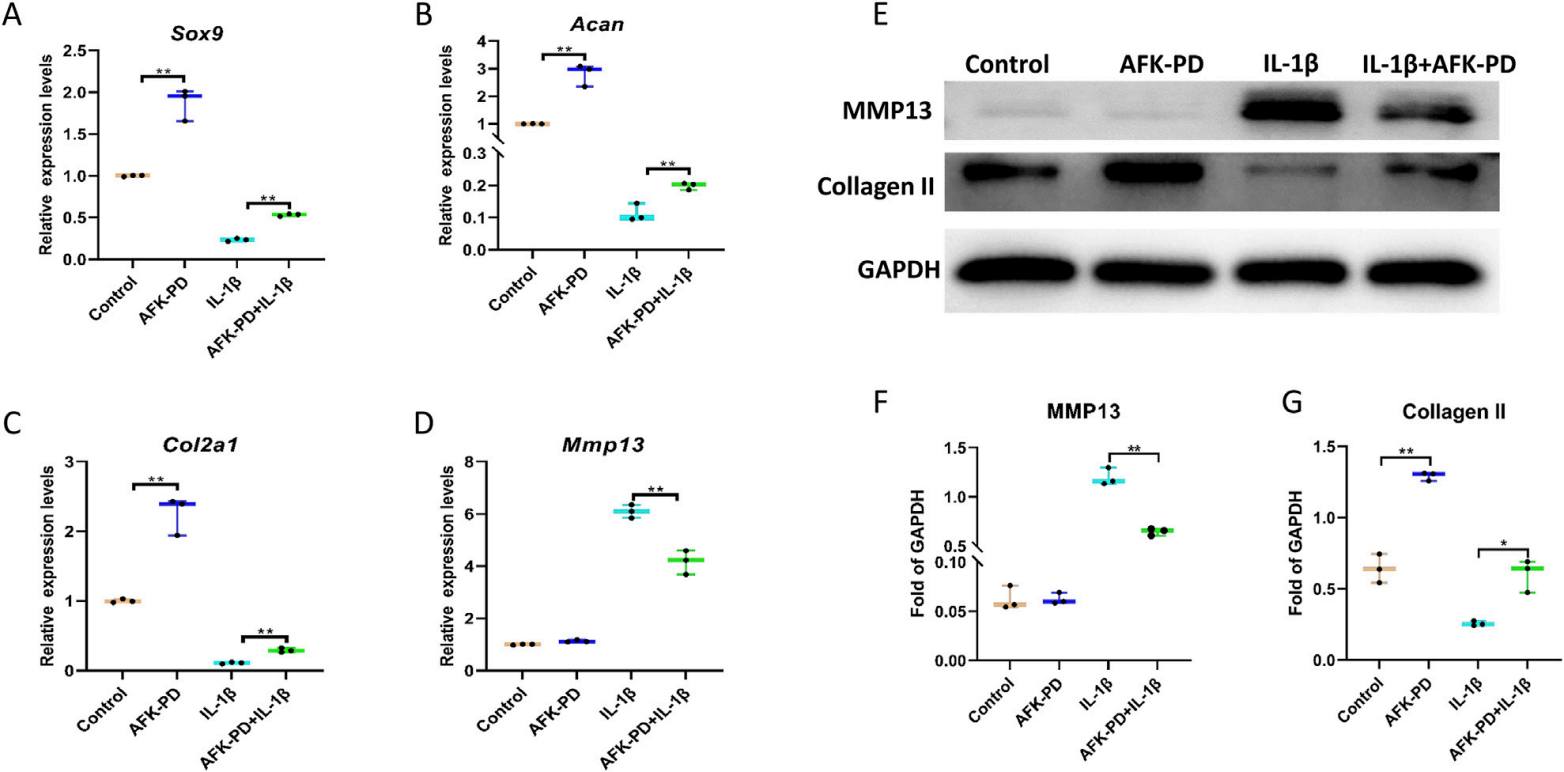
The influence of AFK-PD on chondrocyte anabolism and catabolism in IL-1β-induced primary chondrocyte. Primary chondrocytes were isolated from the femoral condyles and tibial plateau of 3-day-old mice. **(A–D)** RT-qPCR for Sox9, Aggrecan, Col2a1 and Mmp13 in IL-1β-induced primary chondrocyte with or without AFK-PD. **(E)** Western blot for Collagen II and MMP13 in IL-1β-induced primary chondrocyte with or without AFK-PD. And quantitative of Collegan II and Mmp13 was shown on the bottom **(F, G)**. Data are presented as mean ± SD. (n = 3/group, Student t test; *p < 0.05, **p < 0.01).

**FIGURE 2:**
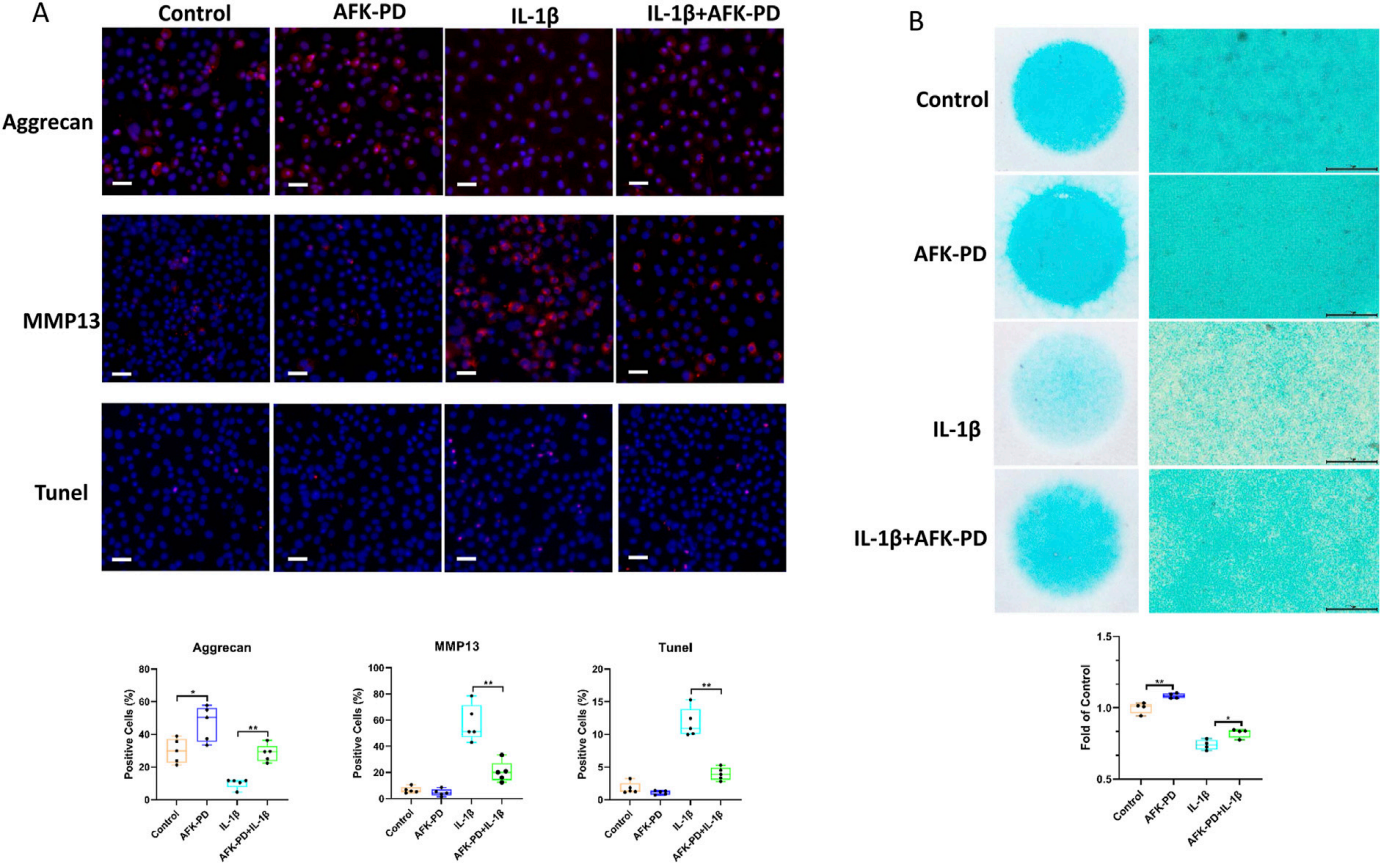
The effects of AFK-PD on chondrocyte anabolism, catabolism and apoptosis in IL-1β-induced primary chondrocyte. **(A)** The alcian blue staining of chondrocyte micro-mass in IL-1β-induced primary chondrocyte with or without AFK-PD for 6 /s (scale bars: 500 μm). The alcian blue staining was quantized on the bottom (n = 4). **(B)** The immunofluorescence for Aggrecan, MMP13 and TUNEL expression in primary chondrocyte IL-1β-induced primary chondrocyte with or without AFK-PD (scale bars: 50 μm). And quantitative of the positive cells was shown on the bottom (n = 5). Data are presented as mean ± SD. (Student t test; *p < 0.05, **p < 0.01).

To clearly study the effect of AFK-PD on chondrocyte’s metabolism, we firstly detected the mRNA expression level of catabolic and anabolic makers in AFK-PD-treated primary chondrocytes using RT-qPCR analysis. The results exhibited the increased expression of anabolic makers (Sox9, Acan and Col2a1) but no markedly difference of catabolic marker Mmp13 in AFK-PD-treated chondrocytes compared to control (Figures 1A–D). Additionally, when primary chondrocytes were induced with IL-1β for 24 h, AFK-PD ameliorated the IL-1β-mediated low expression of anabolic makers and high expression of Mmp13 (Figures 1A–D). We further confirmed the effect of AFK-PD at the protein level. Western blot experiments uncovered AFK-PD increased Collagen II expression but had no effect on MMP13 expression in chondrocytes (Figures 1E–G). After IL-1β intervention, Collagen II expression was inhibited, and MMP13 expression was enhanced. Moreover, AFK-PD rescued the decreased Collagen II and increased MMP13 in chondrocytes induced by IL-1β (Figures 1E–G). Similar results are also observed in immunofluorescence (IF) analysis (Figure 2A). Next, chondrocyte micro-mass cultures were used to assess the contribution of AFK-PD to chondrocyte differentiation. After 6 days of AFK-PD treatment, alcian blue staining displayed a more robust stain in AFK-PD-treated chondrocytes compared to the control. Moreover, AFK-PD improved the reduced stain in IL-1β-induced chondrocytes (Figure 2B).

Because of the important role of chondrocytes apoptosis in OA progression (Hosseinzadeh et al., 2016), we further explored the influence of AFK-PD on apoptosis in chondrocytes with or without IL-1β using TUNEL staining. As showed in Figure 2A, there were no chance observed between AFK-PD and control chondrocytes. However, AFK-PD inhibited the high occurrence of TUNEL-positive cells in IL-1β-induced chondrocytes. The above results indicated AFK-PD promoted chondrocyte’s anabolism, as well as inhibited chondrocyte’s catabolism and apoptosis in IL-1β-induced chondrocytes.

### 3.2 AFK-PD inhibited inflammation in IL-1β-induced chondrocytes

Considering IL-1β as a major inducer of chondrocytes inflammation (Wang et al., 2023), we studied the involvement of AFK-PD in inflammation in IL-1β-induced chondrocytes. RT-qPCR results revealed no significant difference in the mRNA expression of *Inos, Il6* and *Cxcl5* but a decreased expression of *Cox2*, *Il1b* and *Cxcl3* in chondrocytes after AFK-PD treatment. However, AFK-PD obviously inhibited IL-1β-induced high mRNA expression of *Inos*, *Cox2*, *Il6*, *Il1b*, *Cxcl3* and *Cxcl5* in primary chondrocytes (Supplementary Figure S3). These data suggested AFK-PD suppressed IL-1β-induced inflammation in chondrocytes.

### 3.3 AFK-PD restrained MAPK and NF-KB pathways in IL-1β-induced chondrocytes

**FIGURE 3:**
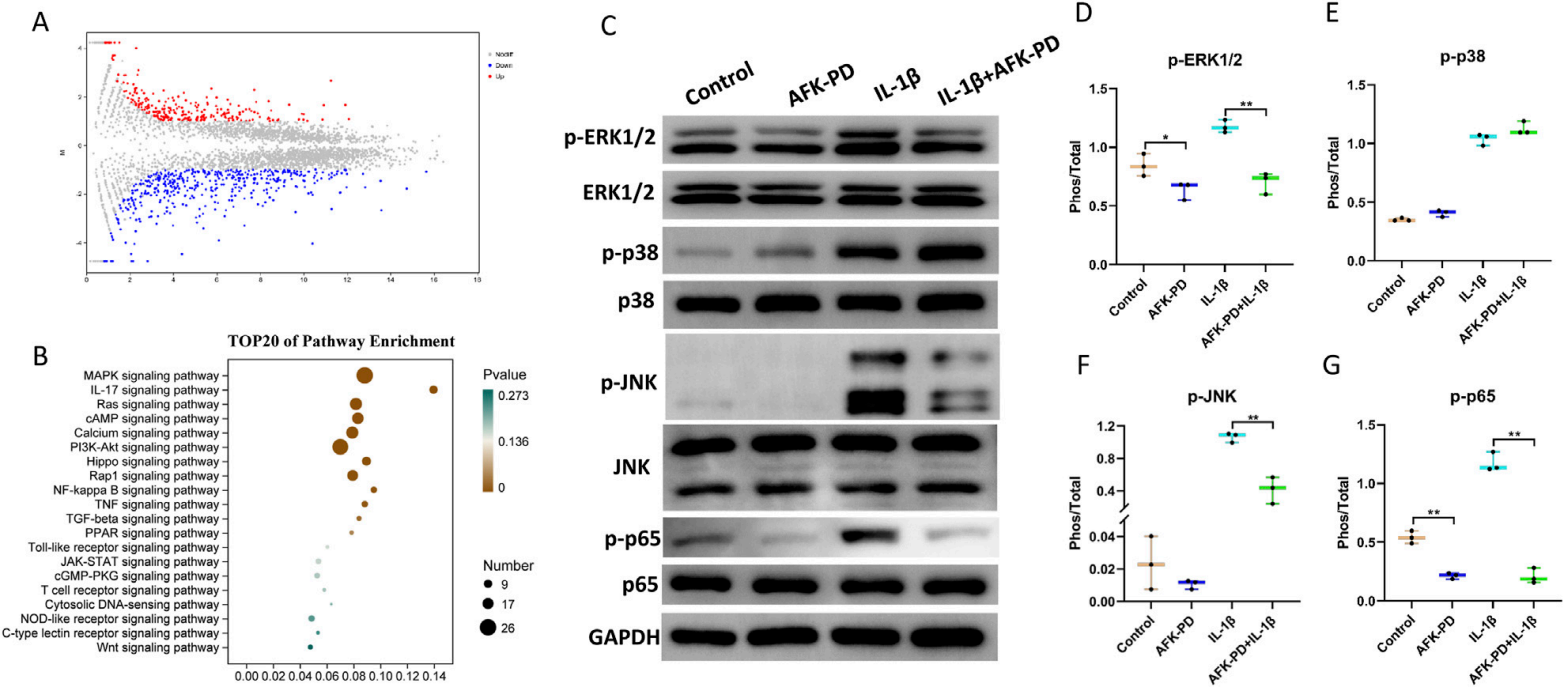
AFK-PD inhibited MAPK and NF-κB pathways in IL-1β-induced primary chondrocytes. **(A)** Volcano plot of RNA-seq analysis for differentially expressed genes between IL-1β-induced primary chondrocyte with and without AFK-PD. **(B)** Bar plot showing the top 20 enriched KEGG pathways in the differentially expressed genes between IL-1β-induced primary chondrocyte with and without AFK-PD. **(C)** Western blot for the protein expression of p-ERK1/2, ERK1/2, p-p38, p38, p-JNK, JNK, p-P65 and p65 in L-1β-induced primary chondrocyte with or without AFK-PD. Quantitative of the protein expression is was shown on the right **(D–G)**. Data are presented as mean ± SD. (n = 3/group, Student t test; *p < 0.05, **p < 0.01).

To further explore the potential molecular mechanisms underlying the effect of AFK-PD on IL-1β-induced chondrocytes function, we performed RNA-sequencing analysis on IL-1β-induced chondrocytes with and without AFK-PD treatment. The volcano plot showed differentially expressed genes (DEGs) between AFK-PD-treated and control chondrocytes in the presence of IL-1β. Among these DEGs, 457 genes were downregulated and 260 genes were upregulated in AFK-PD-treated chondrocytes compared to control chondrocytes (Figure 3A). Furthermore, Kyoto Encyclopedia of Genes and Genomes (KEGG) analysis exhibited the top 20 enrichment signaling pathways (Figure 3B). Notably, the MAPK and NF-κB signaling pathways, which are known to play important roles in chondrocyte differentiation, apoptosis, and inflammation (Lu et al., 2023), were among the identified pathways (Figure 3B).

**FIGURE 4:**
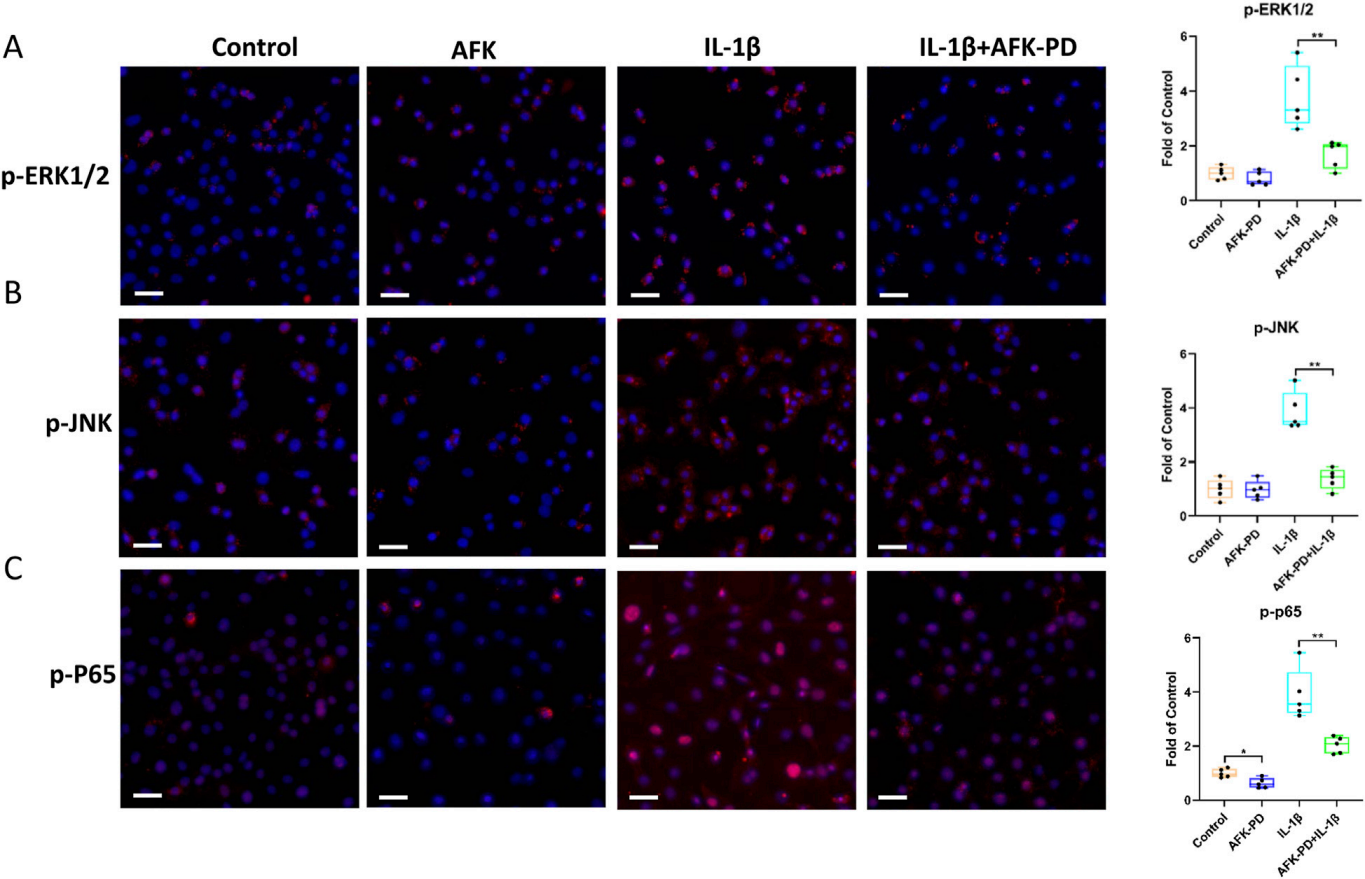
AFK-PD restrained MAPK and NF-κB pathways in IL-1β-induced primary chondrocytes. **(A–C)** The immunofluorescence for p-ERK1/2, p-JNK and p-p65 expression in primary chondrocyte IL-1β-induced primary chondrocyte with or without AFK-PD (scale bars: 50 μm). And quantitative of the positive cells was shown on the right. Data are presented as mean ± SD. (n = 5/group, Student t test; *p < 0.05, **p < 0.01).

First, we examined the expression of key factors in the MAPK signaling pathway, namely, p-ERK1/2, p-JNK, and p-p38, in chondrocytes with or without AFK-PD treatment. Western blot revealed AFK-PD inhibited the protein expression of p-ERK1/2 but had no effect on p-JNK and p-p38. And also, AFK-PD decreased IL-1β-induced high expression of p-ERK1/2 and p-JNK (Figures 3C–F) in chondrocytes. Similar results were confirmed by IF analysis (Figures 4A, B). Next, we assessed the expression of p-p65, a key factor in the NF-κB signaling pathway, using western blot analysis. AFK-PD-treated chondrocytes showed decreased expression of p-p65 compared to the control. Moreover, AFK-PD mitigated the increased expression of p-p65 in chondrocytes induced by IL-1β (Figures 3C, G). Similarly, IF analysis revealed that AFK-PD resulted in decreased expression of p-p65 in chondrocytes with or without IL-1β stimulation (Figure 4C). Hence, it was proposed that AFK-PD inhibited MAPK and NF-κB pathways in IL-1β-induced chondrocytes.

### 3.4 AFK-PD ameliorated the development of DMM-induced OA

**FIGURE 5:**
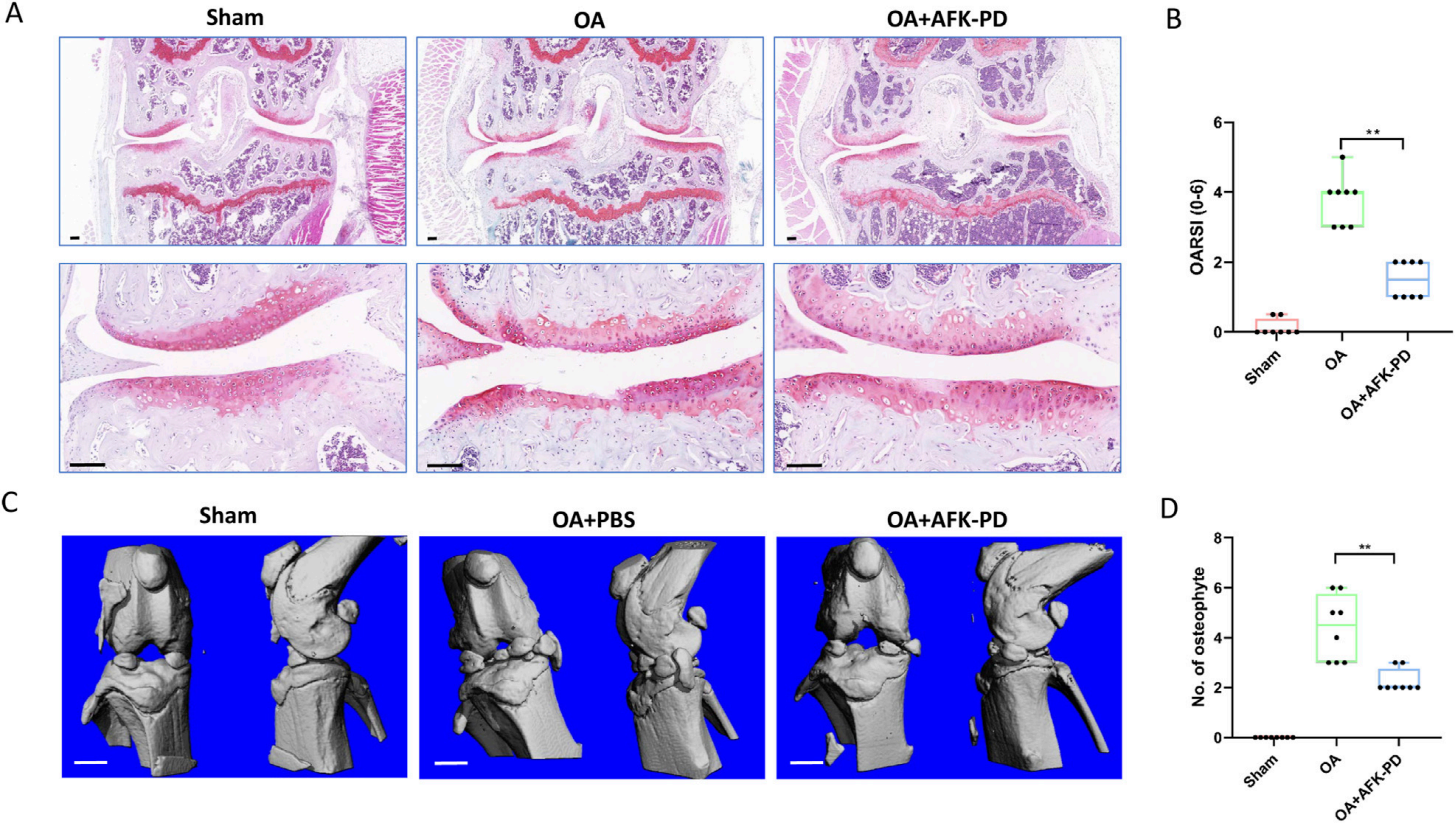
AFK-PD inhibited articular cartilage degradation and osteophyte formation at 8 weeks post-OA surgery. **(A)** The safranin O–fast green staining of knee joint in sham, OA and OA + AFK-PD mice (scale bars: 100 μm). **(B)** OARSI scores of the medial femoral condyle and tibial plateau in sham, OA and OA + AFK-PD mice (n = 8). **(C)** 3D reconstructed images of mice knee joints from sham, OA and OA + AFK-PD mice (scale bars: 1 mm). **(D)** Quantified changes in number of osteophytes. Data are presented as mean ± SD (n = 8/group, Student t test; *p < 0.05, **p < 0.01).

The above results demonstrate that AFK-PD inhibits catabolic metabolism, apoptosis, and inflammation while promoting anabolic metabolism in IL-1β-induced chondrocytes *in vitro*. To further dissect the contribution of AFK-PD to the progression of OA, we conducted *in vivo* experiments. Mice underwent destabilized medial meniscus (DMM) surgery or sham surgery on their right knees. One week post-operation, mice were administrated intra-articular injection of AKK-PD once a week. After 7-week treatment, histological changes of articular cartilage were evaluated using Safranin-O staining and scoring of OARSI grade. Sham-operated mice showed no changes in the articular cartilage, while DMM-operated mice exhibited extensive loss of Safranin-O staining and vertical erosion extending to the calcified cartilage, encompassing over 25% of the area. However, AFK-PD-treated OA mice revealed minimal loss of Safranin-O staining and cartilage (Figure 5A). Further, the OARSI scoring system revealed lower scores in AFK-PD-treated OA mice compared to OA mice (Figure 5B).

Micro-CT was subjected to assess osteophyte formation, a major pathological feature of OA. Sham-operated mice showed no signs of osteophyte formation, while OA mice exhibited numerous osteophytes around the tibial plateau and femoral condyles. Following AFK-PD treatment, the results showed a lower number of osteophytes in AFK-PD-treated OA mice compared to OA mice (Figures 5C, D). So, the above data indicated AFK-PD ameliorated cartilage degeneration and osteophyte formation in OA progression.

### 3.5 Effect of AFK-PD on chondrocyte metabolism and apoptosis in OA cartilage

**FIGURE 6:**
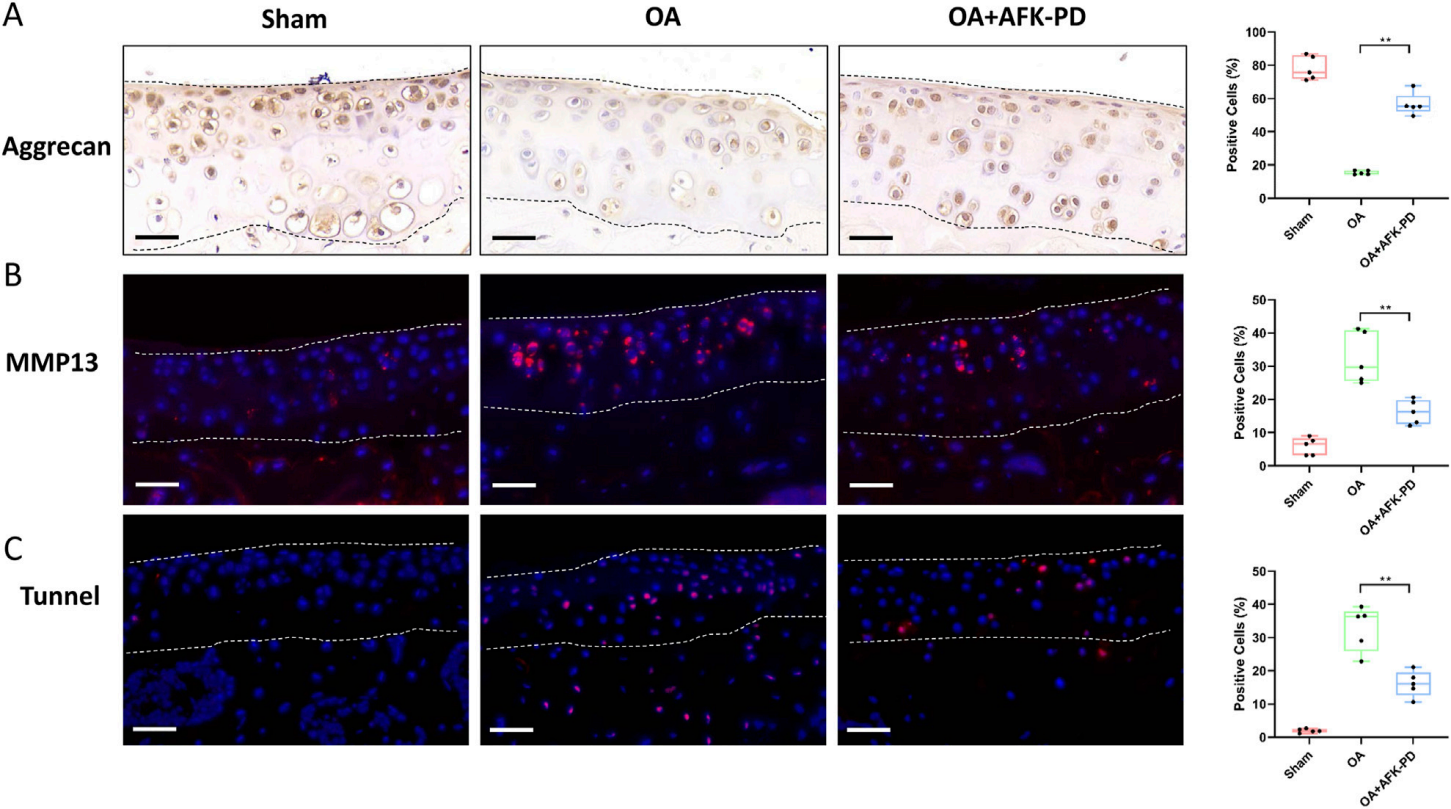
AFK-PD enhanced Aggrecan expression and restrained MMP13 and apoptosis of articular cartilage in mice after DMM surgery. **(A)** The Immunohistochemistry for Aggrecan in the articular cartilage in sham, OA and OA + AFK-PD mice at 8 weeks post OA surgery (scale bars: 50 μm), and quantitative analysis of the positive cells was shown on the right. The articular cartilage was marked between two black dotted lines. **(B)** The immunofluorescence for MMP13 expression in the articular cartilage in sham, OA and OA + AFK-PD mice (scale bars: 50 μm), and quantitative analysis of the positive cells was shown on the right. The articular cartilage was marked between two white dotted lines. **(C)** The immunofluorescence for apoptosis marker TUNEL in the articular cartilage in sham, OA and OA + AFK-PD mice (scale bars: 50 μm), and quantitative analysis of the positive cells was shown on the right. The articular cartilage was marked between two white dotted lines. Data are presented as mean ± SD (n = 5/group, Student t test; *p < 0.05, **p < 0.01).

To further elucidate the cellular mechanism underlying AFK-PD-mediated alleviation of OA progression, we detected the expression of chondrocyte factors related to OA progression. Immunohistochemistry was subjected to assess chondrocyte anabolic factor Aggrecan in articular cartilage. As seen in Figure 6A, the cartilage of OA mice had lower Aggrecan expression than sham mice, but AFK-PD-treated OA mice showed more robust expression of Aggrecan in articular cartilage than OA mice. Next, IF analysis demonstrated an increased expression level of MMP13 in the cartilage of OA mice compared to sham mice. However, AFK-PD treatment rescued the higher expression of MMP13 in the cartilage of OA mice (Figure 6B). Furthermore, we determined the contribution of AFK-PD to chondrocyte apoptosis in cartilage of OA mice using TUNEL staining. The analysis revealed an increased number of TUNEL-positive cells in the cartilage of OA mice compared to sham mice. However, AFK-PD treatment alleviated the enhanced number of TUNEL-positive cells in the cartilage of OA mice (Figure 6C). The above results suggest that AFK-PD ameliorates cartilage degeneration by inhibiting chondrocyte catabolic metabolism and apoptosis while promoting anabolic metabolism.

### 3.6 AFK-PD inhibited synovial inflammation by dampening M1 macrophage polarization

**FIGURE 7:**
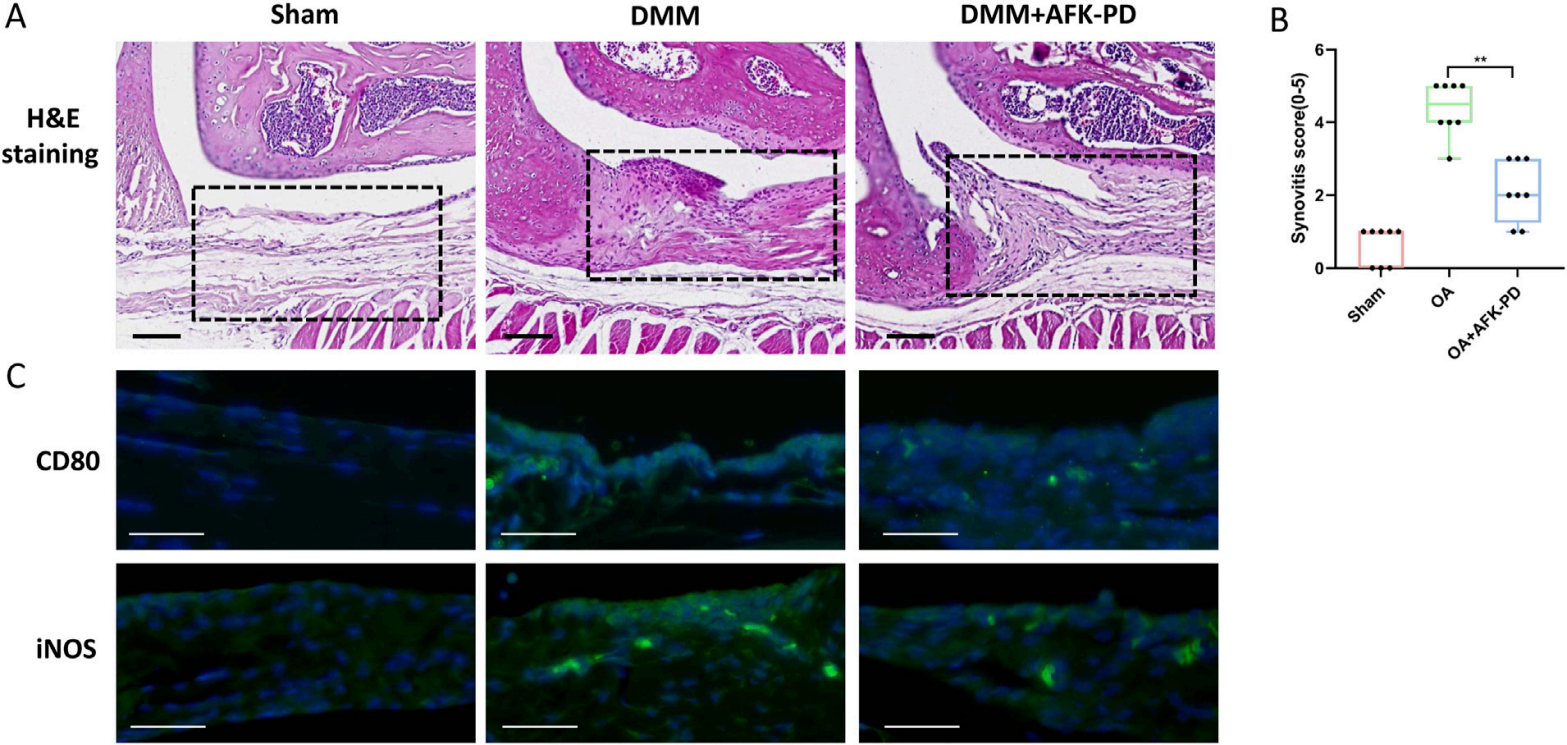
AFK-PD relieved synovitis in OA. **(A)** H&E staining of the synovium in sham, OA, and OA + AFK-PD groups at 8 weeks post OA surgery. Scale bars: 100 μm. Black boxed areas showed synovium. **(B)** Quantification of the synovitis scores of samples was shown on the right (n = 8/group). **(C)** The immunofluorescence for CD80 and iNOS in synovium from sham, OA, and OA + AFK-PD mice (scale bars: 100 μm). Data are presented as mean ± SD (Student t test; *p < 0.05, **p < 0.01).

Considering the vital contribution of synovial inflammation to initiation and progression of OA (Sanchez-Lopez et al., 2022), H&E staining was carried out to assess synovial inflammation. Synovium of OA mice revealed high levels of synovial hyperplasia and abundant cell infiltration, characteristic of synovitis, along with higher synovitis scores compared to sham mice (Figures 7A, B). However, after AFK-PD treatment, a decrease in synovial hyperplasia and cell infiltration was observed along with lower synovitis scores in the synovium compared to OA mice (Figures 7A, B). Synovitis is mainly characterized by enhancing synovial M1 macrophages (pro-inflammation macrophage) (Zhang et al., 2018). Thus, we detected the expression of M1 macrophage markers (CD80 and iNOS) in the synovium using IF. As shown in Figure 7C, the expression of these markers uncovered more robust in synovium of OA mice compared to sham mice, but AFK-PD treatment partially inhibited their high expression.

Above results indicated AFK-PD inhibited M1 macrophage polarization in OA synovium. We further confirmed the effect of AFK-PD on macrophage M1 polarization in RAW264.7 cells induced by LPS. RT-qPCR results revealed AFK-PD had no effect on M1-related markers (*Il6*, *Inos*, *Il1b* and *Mmp13*) in RAW264.7 cells without LPS. However, AFKP-PD attenuated the LPS-induced high mRNA expression of these M1-related markers in RAW264.7 cells (Supplementary Figure S4AD). IF further confirmed AFK-PD rescued the increased protein expression of iNOS in RAW264.7 cells with LPS (Supplementary Figure S4E). These results indicated that AFK-PD suppresses synovial inflammation by inhibiting M1 macrophage polarization.

## 4 Discussion

OA is the most prevalent joint disease characterized by cartilage degeneration and low-grade inflammation. The articular inflammatory environment is the key factor contributing to cartilage degeneration (Sanchez-Lopez et al., 2022). Currently, early-stage OA was widely treated with nonsteroidal anti-inflammatory drugs (NSAIDs) to only symptom relief, but these treatments are unable to prevent cartilage degeneration (Liu-Bryan and Terkeltaub, 2015). Consequently, end-stage OA was often submitted to replacement arthroplasty. To date, no effective and available drugs to prevent and treat OA. Therefore, it is an urgent concern to develop new therapeutic drugs that can effectively prevent the deterioration of joint cartilage in osteoarthritis. In our study, we found AFK-PD, a novel pyridone agent, inhibited IL-1β-induced chondrocyte inflammation. And also, AFK-PD improved synovitis in OA mice by inhibiting M1 macrophages polarization. Similar effects were observed as AFK-PD ameliorated lethal endotoxemia in mice by inhibiting the production of TNF-α and IL-1β in M1 macrophages (Tang et al., 2010). Other studies showed AFK-PD restrained inflammation of renal injury by reducing the expression of chemokines, pro-inflammatory cytokines and NLRP3 inflammasome in mouse peritoneal M1 macrophages (Tang et al., 2015; Liao et al., 2021). AFK-PF also mitigated the inflammation of hepatic cirrhosis by inhibiting peritoneal M1 macrophages. Furthermore, AFK-PD inhibits inflammation in acute lung injury by reducing the number of F4/80-labeled macrophages in mice lungs (Lv et al., 2021). Our results indicated that AFK-PD inhibited both chondrocyte and macrophage-mediated inflammation.

As is widely recognized, the imbalance between chondrocyte catabolic and anabolic metabolism is the direct driver of cartilage degeneration during OA progression (Segarra-Queralt et al., 2024). Therefore, we explored the contribution of AFK-PD to chondrocyte’s metabolism. Without IL-1β interference, AFK-PD enhanced anabolic metabolism and decreased catabolic metabolism in primary chondrocyte. And AFK-PD rescued particially the IL-1β-induced lower anabolic metabolism and higer catabolic metabolism. These results suggested AFK-PD not only regulated chondrocyte’s metabolism under physiological status, but also remodeled imbalance of chondrocyte anabolic and catabolic metabolism induced by inflammation. Based on the AFK-PD-promoted chondrocyte diffirentiation *per se*, we wondered whether AFK-PD promotes the chondrogenesis of mesenchymal stem cell (MSC). This is particularly important if AFK-PD promotes cartilage regeneration derived from MSCs to repair cartilage defects.

Chondrocytes, the sole resident cells in articular cartilage, are required for maintaining cartilage structure and homeostasis. Therefore, the survival of chondrocytes is vital for the normal physiological state of the articular cartilage. It is widely recognized that chondrocyte apoptosis is essential for the occurrence and progression of OA (Hosseinzadeh et al., 2016; Li et al., 2024). In our study, we found AFK-PD inhibited the apoptosis of chondrocyte induced by IL-1β *in vitro*. Furthermore, AFK-PD dampened obviously chondrocyte apoptosis in articular cartilage from OA mice. This finding is consistent with previous evidence demonstrating that AFK-PD alleviated apoptosis in acetaminophen-induced acute liver failure (Gu et al., 2023). And AFK-PD also ameliorated cell apoptosis of kidney in cisplatin-induced acute kidney injury mice and cisplatin-treated NRK-52E cells (Jiang et al., 2019). Moreover, AFK-PD attenuated pulmonary apoptosis in LPS-induced acute lung injury mice (Lv et al., 2021). Therefore, our study further expands our understanding of the anti-apoptotic effects of AFK-PD.

The activation of NF-κB and MAPK pathways are closely involved in aggravation of OA, leading to production of pro-inflammatory cytokines and metaloproteinases both in chondrocyte and synovial macrophage. This ultimately results in imbalance of chondrocyte metabolism and cartilage matrix degeneration (Yan et al., 2020; Gratal et al., 2022; Lu et al., 2023). Our study showed NF-κB and MAPK pathways were significantly activated after stimulation with IL-1β. However, AFK-PD demonstrated the ability to inhibit the phosphorylation level of key factors associated with NF-κB and MAPK pathways. This finding suggests that AFK-PD suppresses chondrocyte inflammation and shifts chondrocyte metabolism from catabolism to anabolism in IL-1β-induced chondrocytes by inhibiting NF-κB and MAPK pathways. Consistent with these results, AFK-PD inhibited inflammation in chronic renal failure and acute kidney injury via mitigating NF-κB and MAPK pathways (Tang et al., 2015; Jiang et al., 2019). Moreover, AFK-PD restrained hepatic inflammation in hepatic cirrhosis by blocking the activation of NF-κB pathways (Tu et al., 2021). Moreover, AFK-PD had anti-inflammation effect on acute lung injury through inhibiting MAPK and NF-κB pathway (Lv et al., 2021).

In conclusion, our findings present AFK-PD as a promising candidate for the treatment of OA. We demonstrated that AFK-PD effectively delayed the development of OA by inhibiting inflammation in chondrocytes and suppressing M1 polarization of synovial macrophages. Furthermore, AFK-PD exhibited positive effects in reducing cartilage degeneration by protecting the chondrocyte functions. Mechanistic investigations revealed that AFK-PD’s effects in IL-1β-induced chondrocytes were mediated through the MAPK and NF-κB pathways. Of note, many of risk factors had involved in initiation and development of OA, including biomechanical injury, aging and obesity. In our study, we focused on assessing the impact of AFK-PD on the progression of traumatic osteoarthritis induced by destabilization of the medial meniscus (DMM), a biomechanical injury. However, the specific contribution of AFK-PD to the initiation and development of aging and obesity-related OA remains unclear. Therefore, it is crucial to expand future studies to evaluate the treatment effects of AFK-PD on OA using mouse models that represent aging and obesity-related OA.

## Data Availability

The original contributions presented in the study are included in the article/Supplementary Material, further inquiries can be directed to the corresponding author/s.

## References

[B1] Atsuta Y. Tomizawa R. R. Levin M. Tabin C. J. (2019). L-type voltage-gated Ca(2+) channel CaV1.2 regulates chondrogenesis during limb development. Proc. Natl. Acad. Sci. U. S. A. 116 (43), 21592–21601. 10.1073/pnas.1908981116 31591237 PMC6815189

[B2] Gerwin N. Bendele A. M. Glasson S. Carlson C. S. (2010). The OARSI histopathology initiative - recommendations for histological assessments of osteoarthritis in the rat. Osteoarthr. Cartil. 18 (Suppl. 3), S24–S34. 10.1016/j.joca.2010.05.030 20864021

[B3] Glasson S. S. Blanchet T. J. Morris E. A. (2007). The surgical destabilization of the medial meniscus (DMM) model of osteoarthritis in the 129/SvEv mouse. Osteoarthr. Cartil. 15 (9), 1061–1069. 10.1016/j.joca.2007.03.006 17470400

[B4] Glasson S. S. Chambers M. G. Van Den Berg W. B. Little C. B. (2010). The OARSI histopathology initiative - recommendations for histological assessments of osteoarthritis in the mouse. Osteoarthr. Cartil. 18 (Suppl. 3), S17–S23. 10.1016/j.joca.2010.05.025 20864019

[B5] Gosset M. Berenbaum F. Thirion S. Jacques C. (2008). Primary culture and phenotyping of murine chondrocytes. Nat. Protoc. 3 (8), 1253–1260. 10.1038/nprot.2008.95 18714293

[B6] Gratal P. Mediero A. Lamuedra A. Matamoros-Recio A. Herencia C. Herrero-Beaumont G. (2022). 6-Shogaol (enexasogoal) treatment improves experimental knee osteoarthritis exerting a pleiotropic effect over immune innate signalling responses in chondrocytes. Br. J. Pharmacol. 179 (22), 5089–5108. 10.1111/bph.15908 35760458

[B7] Gu L. He X. Zhang Y. Li S. Tang J. Ma R. (2023). Fluorofenidone protects against acute liver failure in mice by regulating MKK4/JNK pathway. Biomed. Pharmacother. 164, 114844. 10.1016/j.biopha.2023.114844 37224750

[B8] Hashizume H. Motonari H. Yamamoto K. Nakamura Y. Hisaoka-Nakashima K. Morioka N. (2024). Stimulation of nuclear receptor REV-ERBs alleviates monosodium iodoacetate-induced osteoarthritis pathology of mice and the induction of inflammatory molecules expression in primary cultured chondrocytes. Int. Immunopharmacol. 127, 111349. 10.1016/j.intimp.2023.111349 38086272

[B9] Hosseinzadeh A. Kamrava S. K. Joghataei M. T. Darabi R. Shakeri-Zadeh A. Shahriari M. (2016). Apoptosis signaling pathways in osteoarthritis and possible protective role of melatonin. J. Pineal Res. 61 (4), 411–425. 10.1111/jpi.12362 27555371

[B10] Jiang Y. Quan J. Chen Y. Liao X. Dai Q. Lu R. (2019). Fluorofenidone protects against acute kidney injury. FASEB J. 33 (12), 14325–14336. 10.1096/fj.201901468RR 31661638

[B11] Li J. W. Wang R. L. Xu J. Sun K. Y. Jiang H. M. Sun Z. Y. (2022). Methylene blue prevents osteoarthritis progression and relieves pain in rats via upregulation of Nrf2/PRDX1. Acta Pharmacol. Sin. 43 (2), 417–428. 10.1038/s41401-021-00646-z 33833406 PMC8792025

[B12] Li X. Zhao C. Mao C. Sun G. Yang F. Wang L. (2024). Oleic and linoleic acids promote chondrocyte apoptosis by inhibiting autophagy via downregulation of SIRT1/FOXO1 signaling. Biochim. Biophys. Acta Mol. Basis Dis. 1870 (4), 167090. 10.1016/j.bbadis.2024.167090 38378085

[B13] Liao X. Jiang Y. Dai Q. Yu Y. Zhang Y. Hu G. (2021). Fluorofenidone attenuates renal fibrosis by inhibiting the mtROS-NLRP3 pathway in a murine model of folic acid nephropathy. Biochem. Biophys. Res. Commun. 534, 694–701. 10.1016/j.bbrc.2020.11.017 33220928

[B14] Liu-Bryan R. Terkeltaub R. (2015). Emerging regulators of the inflammatory process in osteoarthritis. Nat. Rev. Rheumatol. 11 (1), 35–44. 10.1038/nrrheum.2014.162 25266449 PMC4374654

[B15] Lou C. Lin C. Wang W. Jiang H. Cai T. Lin S. (2023). Extracts of Oldenlandia diffusa protects chondrocytes via inhibiting apoptosis and associated inflammatory response in osteoarthritis. J. Ethnopharmacol. 316, 116744. 10.1016/j.jep.2023.116744 37295574

[B16] Lu R. Wang Y. G. Qu Y. Wang S. X. Peng C. You H. (2023). Dihydrocaffeic acid improves IL-1β-induced inflammation and cartilage degradation via inhibiting NF-κB and MAPK signalling pathways. Bone Jt. Res. 12 (4), 259–273. 10.1302/2046-3758.124.BJR-2022-0384.R1 PMC10076109 37492935

[B17] Lv X. Yao T. He R. He Y. Li M. Han Y. (2021). Protective effect of fluorofenidone against acute lung injury through suppressing the MAPK/NF-κB pathway. Front. Pharmacol. 12, 772031. 10.3389/fphar.2021.772031 34987397 PMC8721041

[B18] Peng Y. Li L. Zhang X. Xie M. Yang C. Tu S. (2019). Fluorofenidone affects hepatic stellate cell activation in hepatic fibrosis by targeting the TGF-β1/Smad and MAPK signaling pathways. Exp. Ther. Med. 18 (1), 41–48. 10.3892/etm.2019.7548 31258636 PMC6566051

[B19] Peng Y. Yang H. Zhu T. Zhao M. Deng Y. Liu B. (2013). The antihepatic fibrotic effects of fluorofenidone via MAPK signalling pathways. Eur. J. Clin. Invest 43 (4), 358–368. 10.1111/eci.12053 23438945

[B20] Qian Z. Gao X. Jin X. Kang X. Wu S. (2023). Cartilage-specific deficiency of clock gene Bmal1 accelerated articular cartilage degeneration in osteoarthritis by up-regulation of mTORC1 signaling. Int. Immunopharmacol. 115, 109692. 10.1016/j.intimp.2023.109692 36628892

[B21] Qin J. Mei W. J. Xie Y. Y. Huang L. Yuan Q. J. Hu G. Y. (2015). Fluorofenidone attenuates oxidative stress and renal fibrosis in obstructive nephropathy via blocking NOX2 (gp91phox) expression and inhibiting ERK/MAPK signaling pathway. Kidney Blood Press Res. 40 (1), 89–99. 10.1159/000368485 26029782

[B22] Saklatvala J. (2007). Inflammatory signaling in cartilage: MAPK and NF-kappaB pathways in chondrocytes and the use of inhibitors for research into pathogenesis and therapy of osteoarthritis. Curr. Drug Targets 8 (2), 305–313. 10.2174/138945007779940115 17305508

[B23] Salvat C. Pigenet A. Humbert L. Berenbaum F. Thirion S. (2005). Immature murine articular chondrocytes in primary culture: a new tool for investigating cartilage. Osteoarthr. Cartil. 13 (3), 243–249. 10.1016/j.joca.2004.11.008 15727891

[B24] Sanchez-Lopez E. Coras R. Torres A. Lane N. E. Guma M. (2022). Synovial inflammation in osteoarthritis progression. Nat. Rev. Rheumatol. 18 (5), 258–275. 10.1038/s41584-022-00749-9 35165404 PMC9050956

[B25] Segarra-Queralt M. Crump K. Pascuet-Fontanet A. Gantenbein B. Noailly J. (2024). The interplay between biochemical mediators and mechanotransduction in chondrocytes: unravelling the differential responses in primary knee osteoarthritis. Phys. Life Rev. 48, 205–221. 10.1016/j.plrev.2024.02.003 38377727

[B26] Tang Y. Li B. Wang N. Xie Y. Wang L. Yuan Q. (2010). Fluorofenidone protects mice from lethal endotoxemia through the inhibition of TNF-alpha and IL-1beta release. Int. Immunopharmacol. 10 (5), 580–583. 10.1016/j.intimp.2010.02.005 20159052

[B27] Tang Y. Zhang F. Huang L. Yuan Q. Qin J. Li B. (2015). The protective mechanism of fluorofenidone in renal interstitial inflammation and fibrosis. Am. J. Med. Sci. 350 (3), 195–203. 10.1097/MAJ.0000000000000501 26035627

[B28] Tu S. Jiang Y. Cheng H. Yuan X. He Y. Peng Y. (2021). Fluorofenidone protects liver against inflammation and fibrosis by blocking the activation of NF-κB pathway. FASEB J. 35 (7), e21497. 10.1096/fj.202002402R 34152015

[B29] Wang L. Xu H. Li X. Chen H. Zhang H. Zhu X. (2023). Cucurbitacin E reduces IL-1β-induced inflammation and cartilage degeneration by inhibiting the PI3K/Akt pathway in osteoarthritic chondrocytes. J. Transl. Med. 21 (1), 880. 10.1186/s12967-023-04771-7 38049841 PMC10696753

[B30] Wei Q. Kong N. Liu X. Tian R. Jiao M. Li Y. (2021). Pirfenidone attenuates synovial fibrosis and postpones the progression of osteoarthritis by anti-fibrotic and anti-inflammatory properties *in vivo* and *in vitro* . J. Transl. Med. 19 (1), 157. 10.1186/s12967-021-02823-4 33874948 PMC8054406

[B31] Yan Z. Lin Z. Wu Y. Zhan J. Qi W. Lin J. (2020). The protective effect of myricitrin in osteoarthritis: an *in vitro* and *in vivo* study. Int. Immunopharmacol. 84, 106511. 10.1016/j.intimp.2020.106511 32361653

[B32] Zhang H. Cai D. Bai X. (2020). Macrophages regulate the progression of osteoarthritis. Osteoarthr. Cartil. 28 (5), 555–561. 10.1016/j.joca.2020.01.007 31982565

[B33] Zhang H. Lin C. Zeng C. Wang Z. Wang H. Lu J. (2018). Synovial macrophage M1 polarisation exacerbates experimental osteoarthritis partially through R-spondin-2. Ann. Rheum. Dis. 77 (10), 1524–1534. 10.1136/annrheumdis-2018-213450 29991473

[B34] Zhou F. Mei J. Han X. Li H. Yang S. Wang M. (2019). Kinsenoside attenuates osteoarthritis by repolarizing macrophages through inactivating NF-κB/MAPK signaling and protecting chondrocytes. Acta Pharm. Sin. B 9 (5), 973–985. 10.1016/j.apsb.2019.01.015 31649847 PMC6804452

