# Supplementary material for: AFK-PD alleviated osteoarthritis progression by chondroprotective and anti-inflammatory activity
Source: Front Pharmacol. 2024 Aug 29;15:1439678. doi: 10.3389/fphar.2024.1439678 (PMC11390510; doi:10.3389/fphar.2024.1439678)
Supplement: Supplementary file 1 [file DataSheet1.docx]

**AFK-PD alleviated osteoarthritis progression by** **chondroprotective and anti-inflammatory activity**

Zhuang Qian ^1^†, Jie Xu^1^†, Lei Zhang^1^†, Qian Deng^1^, Zhenlin Fan^1^, Xueqiang Guo^1^, Zhuo Liang^1^, Weiyun Wang^1^, Lei Wang^1*^, Xiaohua Liao^2*^ and Wenjie Ren^1*^

**Supplemental data**

**Supplemental Table 1 Antibodies information**

| Antibody Names | Application and Dilution | Company | Product No. |
| --- | --- | --- | --- |
| GAPDH | WB (1:10000) | Zen-bioscience | 301341 |
| CD80 | IF (1:200) | Proteintech | 66406-1-Ig |
| iNOS | IF (1:300) | Cell Signaling Technology | 13120S |
| MMP13 | WB(1:500),IF(1:200) | Proteintech | 18165-1-AP |
| Collagen II | WB(1:200) | Arigo Biolaboratories | ARG20787 |
| Aggrecan | IF(1:200), IHC(1:300) | Proteintech | 13880-1-AP |
| ERK1/2 | WB (1:1000) | Cell Signaling Technology | 9926T |
| p-ERK1/2 | WB(1:1000),IF(1:2000) | Cell Signaling Technology | 9910T |
| P38 | WB (1:1000) | Cell Signaling Technology | 9926T |
| p-P38 | WB(1:1000) | Cell Signaling Technology | 9910T |
| JNK | WB(1:1000) | Cell Signaling Technology | 9926T |
| p-JNK | WB(1:1000),IF(1:100) | Cell Signaling Technology | 9910T |
| P65 | WB(1:1000) | Cell Signaling Technology | 8242S |
| p-P65 | WB(1:1000),IF(1:800) | Cell Signaling Technology | 3033S |

**Supplemental Table 2 Primer sequences used for RT-qPCR**

| Gene name | Forward (5’-3’) | Reverse(5’-3’) |
| --- | --- | --- |
| *Gapdh* | AGGTCGGTGTGAACGGATTTG | GGGGTCGTTGATGGCAACA |
| *Il6* | CTGCAAGAGACTTCCATCCAG | AGTGGTATAGACAGGTCTGTTGG |
| *Inos* | GTTCTCAGCCCAACAATACAAGA | GTGGACGGGTCGATGTCAC |
| *Il1b* | GAAATGCCACCTTTTGACAGTG | TGGATGCTCTCATCAGGACAG |
| *Mmp13* | TGTTTGCAGAGCACTACTTGAA | CAGTCACCTCTAAGCCAAAGAAA |
| *Cd206* | CTCTGTTCAGCTATTGGACGC | CGGAATTTCTGGGATTCAGCTTC |
| *Arg1* | CTCCAAGCCAAAGTCCTTAGAG | AGGAGCTGTCATTAGGGACATC |
| *Igf1* | CACATCATGTCGTCTTCACACC | GGAAGCAACACTCATCCACAATG |
| *Mrc1* | CTCTGTTCAGCTATTGGACGC | CGGAATTTCTGGGATTCAGCTTC |
| *Sox9* | AGTACCCGCATCTGCACAAC | ACGAAGGGTCTCTTCTCGCT |
| *Col2a1* | GGGTCACAGAGGTTACCCAG | ACCAGGGGAACCACTCTCAC |
| *Acan* | GTGGAGCCGTGTTTCCAAG | AGATGCTGTTGACTCGAACCT |
| *Cox2* | TGAGCAACTATTCCAAACCAGC | GCACGTAGTCTTCGATCACTATC |
| *Cxcl3* | TTGGTGGCAGCTGTGATAGT | TGTGCACATGCATTCTTCCT |
| *Cxcl5* | GTTCCATCTCGCCATTCATGC | GCGGCTATGACTGAGGAAGG |


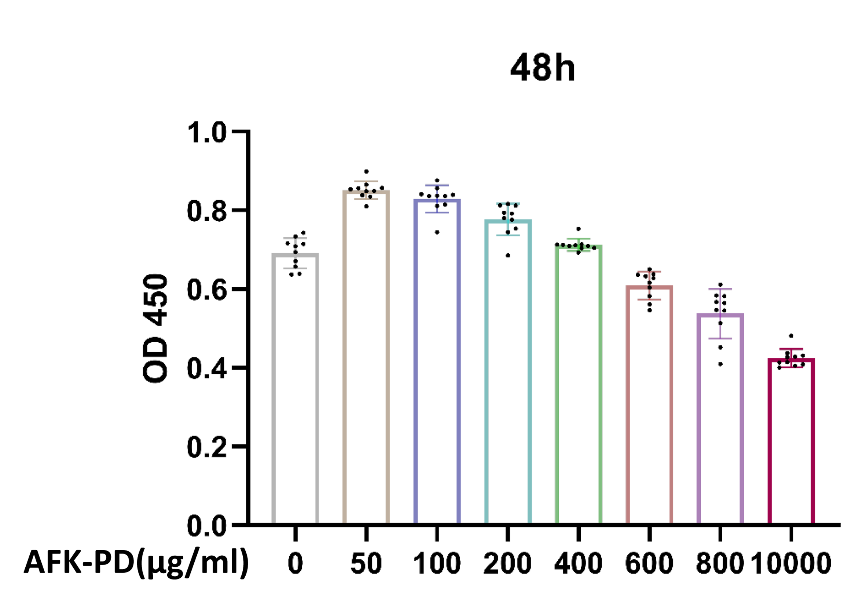


**Supplemental Figure 1. The effect of AFK-PD on cell viability.** Primary chondrocyte was isolated from the femoral condyles and tibial plateau of 3-day-old neonatal mice. CCK-8 assay of cell viability in primary chondrocyte treated with different concentration AFK-PD for 48h. Data are presented as mean ± SD (n=10/group, Student t test; *P < 0.05, **P < 0.01).


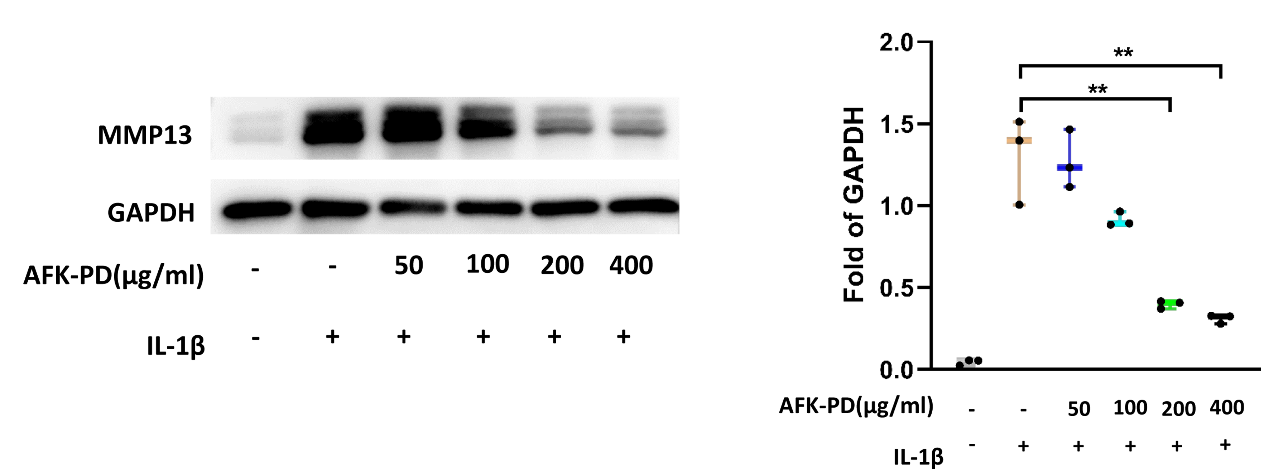


**Supplemental Figure 2. The effect of AFK-PD on MMP13 expression in primary chondrocyte treated with IL-1β.** Primary chondrocyte was isolated from the femoral condyles and tibial plateau of 3-day-old neonatal mice. Western blot for MMP13 in primary chondrocyte treated with IL-1β in treatment of different concentration AFK-PD for 48h. And quantitative of Mmp13 was shown on the right. Data are presented as mean ± SD. (n=3/group, Student t test; *P < 0.05, **P < 0.01).


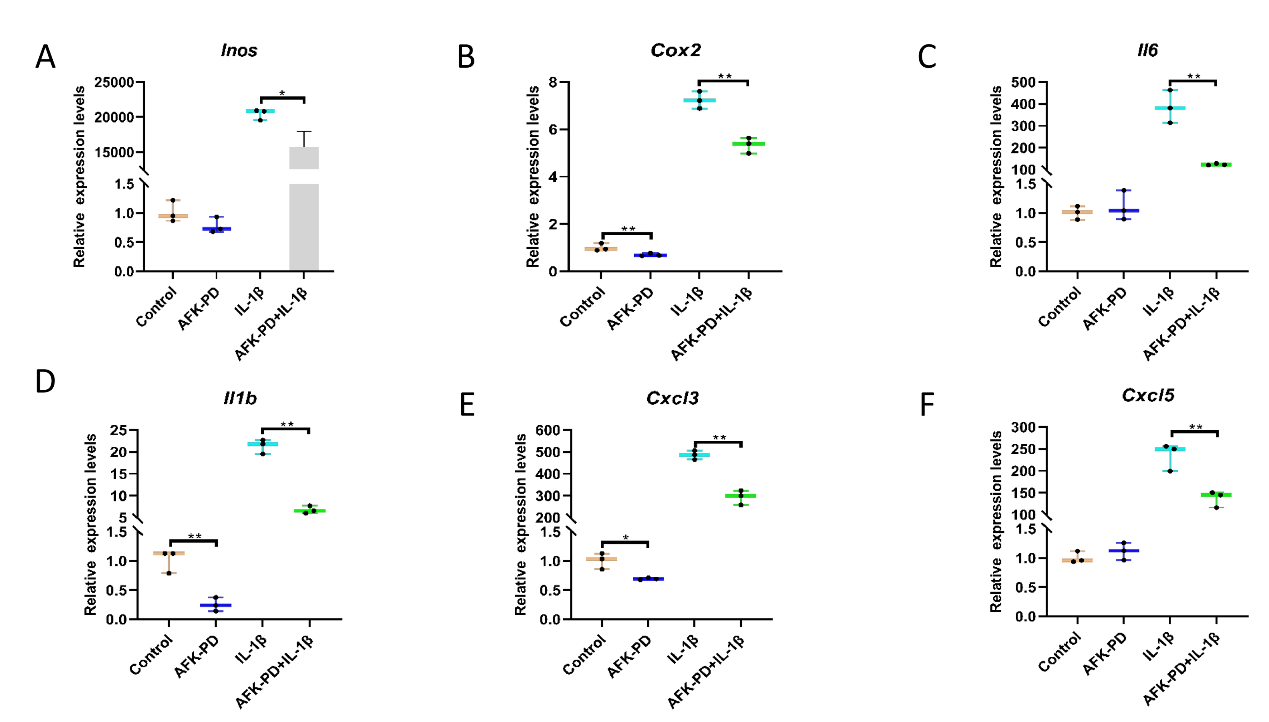


**Supplemental Figure 3.** **AFK-PD restrained chondrocyte inflammation of IL-1β-induced primary chondrocyte.** (A-F) RT-qPCR for inflammation factor (Inos, Cox2, Il6, Il1β, Cxcl3 and Cxcl5) in IL-1β-induced primary chondrocyte with or without AFK-PD. Data are presented as mean ± SD. (n=3/group, Student t test; *P < 0.05, **P < 0.01).


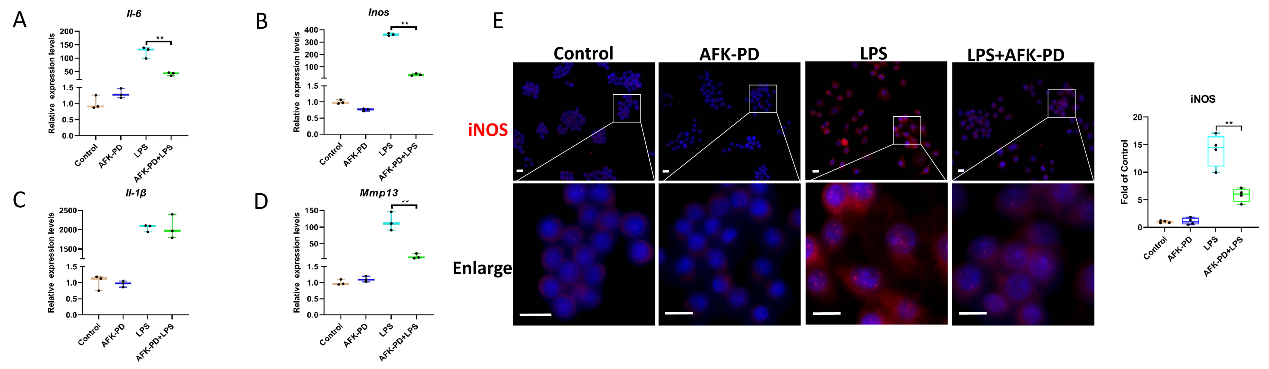


**Supplemental Figure 4. AFK-PD inhibited M1 polarization of macrophage inRAW264.7 cells .** (A-D) RT-qPCR for M1 markers (Il-6, Inos, Il1β and Mmp13) in RAW264.7 cells treated with LPS with or without AFK-PD for 24h. (E) The immunofluorescence for iNOS in RAW264.7 cells treated with LPS with or without AFK-PD for 24h (scale bars: 25μm). And quantitative analysis of the positive cells (red) was shown on the right (n=4). Data are presented as mean ± SD (Student t test; **P* < 0.05, ***P* < 0.01).

**Original Western blots**

**
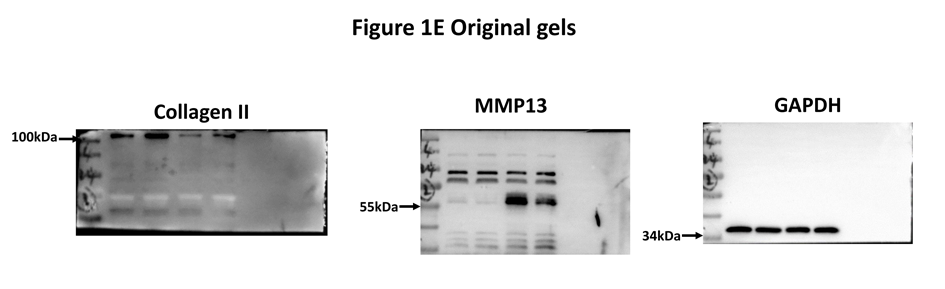
**

**
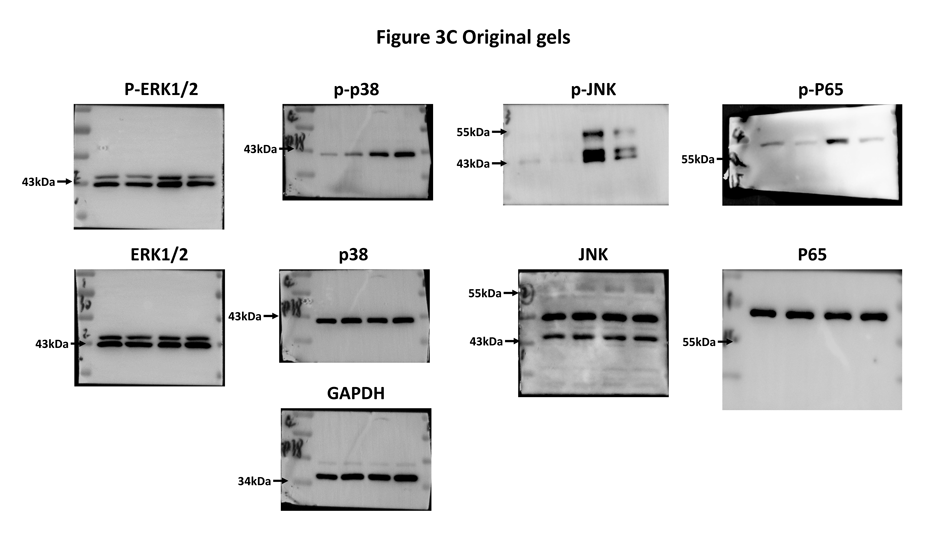
**

**
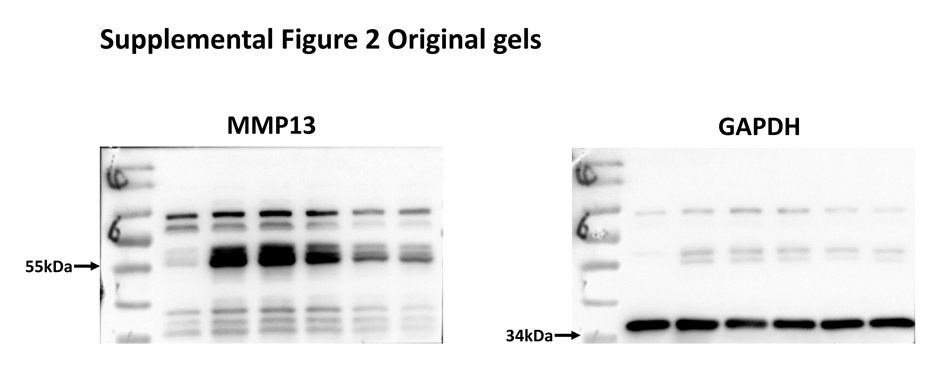
**
